# Immobilization of β-Galactosidase From *Aspergillus oryzae* on Electrospun Gelatin Nanofiber Mats for the Production of Galactooligosaccharides

**DOI:** 10.1007/s12010-020-03252-7

**Published:** 2020-01-24

**Authors:** Ann-Cathérine Sass, Hans-Joachim Jördening

**Affiliations:** Institute of Technical Chemistry, Department of Carbohydrate Technology, Technische Universität Braunschweig, Gaußstraße 17, 38106 Braunschweig, Germany

**Keywords:** Electrospinning, Gelatin, β-Galactosidase, Immobilization, Galactooligosaccharides, Transgalactosylation, *Aspergillus oryzae*

## Abstract

Two simple and easily reproducible methods for the immobilization of β-galactosidase (β-gal) from *Aspergillus oryzae* on electrospun gelatin nanofiber mats (GFM) were developed. The process was optimized regarding the electrospinning solvent system and the subsequent cross-linking of GFM in order to increase their stability in water. β-Gal was covalently immobilized on activated gelatin nanofiber mats with hexamethylenediamine (HMDA) as a bifunctional linker and secondly via entrapment into the gelatin nanofibers during the electrospinning process (suspension electrospinning). Optimal immobilization parameters for covalent immobilization were determined to be at pH 7.5, 40 °C, β-gal concentration of 1 mg/mL and immobilization time of 24.5 h. For suspension electrospinning, the optimal immobilization parameters were identified at pH 4.5 and β-gal concentration of 0.027 wt.% in the electrospinning solution. The pH and temperature optima of immobilized β-gal shifted from 30 °C, pH 4.5 (free enzyme) to pH 3.5, 50 °C (covalent immobilization) and pH 3.5, 40 °C (suspension electrospinning). Striking differences in the Michaelis constant (K_M_) of immobilized β-gal compared with free enzyme were observed with a reduction of K_M_ up to 50% for immobilized enzyme. The maximum velocity (v_max_) of immobilization by suspension electrospinning was almost 20 times higher than that of covalent immobilization. The maximum GOS yield for free β-gal was found to be 27.7% and 31% for immobilized β-gal.

## Introduction

Electrospinning is a versatile and reasonable method for generating continuous nanofibers from a variety of natural or synthetic polymers with diameters ranging from a few micrometers to several nanometers. The standard electrospinning setup consists of a spinneret, which is connected to a high-voltage power supply and of a collector, which is functioning as the counter electrode. When applying an electric field, the electrospinning solution becomes charged. Mutual electric repulsion antagonizes the surface tension of the solution. After reaching a critical value of the electric field, the electric repulsion overcomes the surface tension and a liquid droplet breaks out forming a typical conic shape, known as the Taylor cone. The solvent from the traveling jet evaporates quickly on its way from the tip of the spinneret to the collector, and the jet undergoes simultaneously complex structure forming processes, which result in a tremendous fiber stretch on a proportionally short distance [1, 2]*.* The newly formed nanofibers are randomly collected onto the collector. Electrospun nanofiber mats have many advantages, e.g., an enormous surface-to-mass ratio, extensive convertibility, easy handling, and the malleability to conform fiber diameters, shapes, and pore structures. Electrospun nanofiber mats could also be an excellent material for the immobilization of enzymes since fine nanofiber mats only have little diffusion limitations and offer a big specific surface with plenty of space for chemical bonding or inclusion of great enzyme quantities.

Galactooligosaccharides (GOS) are an inhomogeneous, mixed class of lactose-derived saccharides consisting of two to eight saccharide units and a terminal glucose residue. GOS are contained in the milk of nearly all mammals and feature a prebiotic effect, which influences the composition of the gastrointestinal microflora that assigns benefits to the host health and well-being. Currently, GOS are mainly produced from lactose via a transgalactosylation reaction by the enzyme β-galactosidase (EC 3.2.1.23), where the galactosyl moiety of the substrate lactose is transferred to a nucleophilic acceptor to generate a mixture of oligosaccharides with a huge diversity in structure. Transgalactosylation is kinetically controlled due to the competition between transgalactosylation and the thermodynamically favored hydrolysis of lactose. To make the production of GOS more profitable, it is advantageous to immobilize β-galactosidase in order to recover and reuse the enzyme multiple times. For the choice of a suitable carrier material, it should be considered that diffusional resistances in the carrier material cause a reduction in GOS production due to concentration gradients and a boosted hydrolyzation activity [3, 4]. For the industry, the immobilization of the common β-galactosidase has been a matter of particular interest for a number of years and has been investigated for various materials. β-Galactosidase from *Aspergillus oryzae* was already immobilized, e.g., on colloidal liquid aphrons, on grafted nylon membranes, on cotton cloth, via entrapment in alginate and gelatin fibers, on thermally stable carrageenan coated with chitosan, and onto Duolite A568 resin [5–10]. The immobilization of β-galactosidase on membranes was also reported previously [11, 12]. Surface-bound enzymes are in close proximity to the feed solution thus reducing diffusion limitations and improving the access of a substrate to the active site of the enzyme [13]. The immobilization of enzymes onto electrospun fiber mats as the carrier material is very promising and was carried out by several working groups for different enzymes and polymers [14–18]. The aim of this study is to develop an easy, efficient, and economically viable immobilization procedure for the immobilization of β-galactosidase from *Aspergillus oryzae* on electrospun gelatin nanofiber mats via covalent binding and encapsulation. Immobilized β-gal should be suitable for the production of GOS and for the implementation in continuous large-scale processes.

## Material and Methods

### Material

β-Galactosidase (3.2.1.23) from *Aspergillus oryzae* (> 8.0 units/mg solid), gelatin from porcine skin (type A), and lactose monohydrate were obtained from Sigma (Germany). Sodium tetraborate decahydrate was purchased from abcr GmbH (Germany). All other chemicals were obtained from Carl Roth (Germany).

### Methods

#### Electrospinning of Gelatin Fiber Mats

**Fig. 1:**
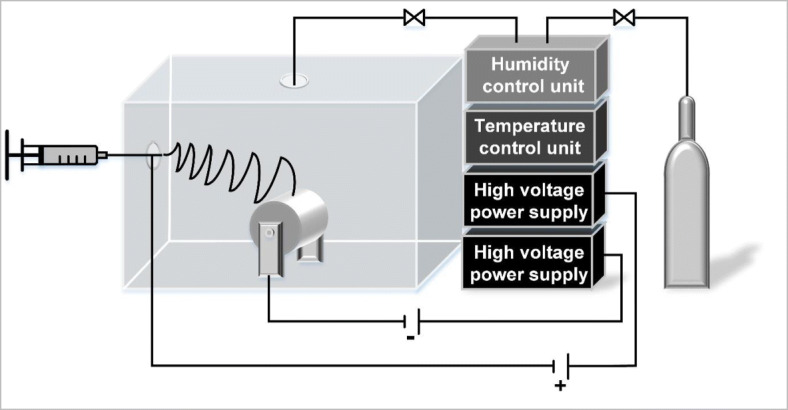
Schematic horizontal electrospinning setup in a climate control chamber with external humidity and temperature regulation. The electrospinning solution is delivered into the chamber by a syringe pump. Nanofibers are captured by a rotational, cylindrical collector with adjustable rotational speed

Gelatin solution (10 wt.%) was prepared in a mixed solvent system of 2,2,2-trifluoroethanol (TFE) and 0.02 mol/L citrate-phosphate buffer (pH 4.5) in varied solvent ratios. The solution was placed in a syringe pump, and the electrospinning was carried out in a self-built climate control chamber (Fig. 1). The rotational collector (1000 rpm) consisted of an aluminum cylinder (Ø 10 cm), which was wrapped with a synthetic fiber mesh. Two adjustable high voltage power supplies were used to supply high DC voltage. The positive electrode (15 kV) was directly clamped on a blunt-ended cannula connected to the syringe, and the negative electrode (7.5 kV) was attached to the collector. The gelatin solution was constantly delivered into the chamber at 1 mL/h. The distance between the needle tip and the collector was 20 cm. The temperature inside the chamber was fixed at 25 °C, and the humidity was set to 30%.

#### Covalent Immobilization of β-Galactosidase Onto GFM With Hexamethylenediamine as a Bifunctional Linker

##### Cross-Linking of GFM

GFM were placed in a sealed glass container with 10 mL of a 25% glutaraldehyde solution (GDA) in a Petri dish to create a GDA atmosphere [19]. Cross-linking was carried out at room temperature for 1–24 h.

##### Activation of Cross-Linked GFM With EDC/NHS and HMDA

Activation solution was prepared by dissolving 200 mmol/L 1-ethyl-3-(3-dimethylaminopropyl)carbodiimide-hydrochloride (EDC) and 40 mmol/L N-hydroxysuccinimide (NHS) in 0.05 mol/L MES buffer (pH 5.3). GFM were placed in 500 μL of activation solution for 70 min at room temperature under slow stirring. The mats were washed in 5 mL of methanol for 30 min to remove isourea byproducts. GFM were immersed in 1 mL of 1 mg/mL HMDA in 0.1 M potassium phosphate buffer (pH 7.5) for 16 h at room temperature.

##### Immobilization of β-Galactosidase on HMDA-Activated GFM

A 1% GDA in 0.2 M sodium borate buffer (pH 8.5) was used to establish a bifunctional linker between HMDA and β-gal. Reaction for each mat was carried out in 1 mL of the GDA solution. After 20 min, the mats were washed for 30 min in ultrapure water. After washing, the mats were immersed in 2 mL of 0.1–5 mg/mL β-galactosidase in 0.02 M citrate phosphate buffer (pH 2.5–8.5) at 8–50 °C for 0–24.5 h. After the incubation, hmdaGFM were washed with 0.02 M citrate-phosphate buffer (pH 4.5) and stored in the same buffer at 4 °C.

#### Direct Immobilization of β-Galactosidase Onto GFM During the Electrospinning Process (Suspension Electrospinning)

β-Galactosidase (0.027 wt.%) was dissolved in a mixed solvent system of 0.02 M citrate phosphate buffer (pH 4.5) and TFE with a ratio of (70:30). Gelatin (10 wt.%) was added to obtain the actual electrospinning solution. Electrospinning was carried out as described previously. After suspension electrospinning, the suspension gelatin fiber mats (suspGFM) were cross-linked under GDA vapor at room temperature for 1–24 h.

#### Characterization of hmdaGFM and suspGFM

The morphology of electrospun gelatin nanofiber mats was analyzed under a scanning electron microscope (Zeiss Evo LS 25). Fiber diameters were determined via the image-processing program ImageJ.

#### Activity Determination via β-Gal Activity Assay

Free β-gal activity was measured using 500 μL 10 mmol/L ortho-nitrophenyl-β-galactoside (oNPG, oNPG assay) or 156 mmol/L lactose in 0.02 M citrate phosphate buffer (pH 4.5, lactose assay). The reaction mixture was pre-heated at 30 °C on a thermo shaker for about 1 min. Then, 0.4 U free β-galactosidase or a 1 × 2 cm rectangle of hmdaGFM or suspGFM was added and incubated for exactly 10 min. Reaction was stopped by 1.5 mL 0.2 M borate buffer (pH 10) (oNPG assay) or by heating samples to 100 °C for 5 min (lactose assay). Then, 2 μL of the sample was measured by NanoDrop 2000 (Thermo Fisher Scientific, Germany) against a suitable blank or samples were analyzed by HPLC.

#### Operational Stability of Free and Immobilized β-Galactosidase

The operational stability of immobilized β-gal on hmdaGFM and suspGFM was studied in 584 mmol/L lactose solution (pH 3.5) at 30 °C under estimating the half-life of β-gal. Both hmdaGFM and suspGFM were stored in 2 mL of the lactose solution at 30 °C in a thermo shaker. At defined time intervals, GFM were withdrawn, and the remaining enzymatic activity was tested via a lactose activity assay. The remaining activity was defined as the release of glucose per minute and per g_GFM_.

#### Kinetic Studies on the Activity of Free and Immobilized β-Galactosidase

The effect of the oNPG concentration on the activity of free and immobilized β-gal was determined by following the production rate of o-nitrophenol over a period of 240 min. For all tests, the oNPG concentration was varied between 1 and 50 mmol/L at 30 °C and pH 4.5 (free β-gal) and 40 °C and pH 3.5 (immobilized β-gal), respectively.

Experimental data from kinetic tests were associated with the Michaelis–Menten equation in the software ModelMaker 3.0 (Cherwell Scientific Inc.). Michaelis–Menten parameters v_max_ and K_M_ were systematically adjusted to reduce the deviation between the model and experimental data. In ModelMaker, simple analytical methods, such as linear regression, are not generally applicable. Therefore, iterative numerical optimization was used (Levenberg–Marquardt algorithm) in combination with Runge-Kutta 4th-order method) for determination of K_M_ and v_max_. The coefficient of determination was defined to be at least 90%.

#### HPLC

Samples containing lactose as the substrate were analyzed by HPLC equipped with a refractive index detector (Shodex RI-501), a Phenomenex SecurityGuard cartridge (Carbo-Ca 4 × 3.0 mm) and a Shodex SC1011 column connected in series at 80 °C. Samples were eluated in degassed ultrapure water at a flow rate of 0.6 mL/min. Concentration of all saccharides was calculated via interpolation of external standards by Clarity Chromatography Station (DataApex, Czech Republic).

## Results and Discussion

### Characterization of Electrospun Gelatin Nanofiber Mats

For the immobilization of β-gal onto electrospun gelatin fiber mats, an adequate solvent system had to be determined (a) to be able to dissolve β-gal evenly in the electrospinning solution without a rigorous reduction in the enzymatic activity (particularly with regard to suspension electrospinning) and (b) to create a solvent system that provides an easy-to-spin gelatin solution resulting in small-diameter fiber mats. With the introduction of an aqueous part into the prior mono-solvent system (TFE), the homogenous dissolving of β-gal was simply attainable even at higher enzyme concentration levels. In addition, the presence of water in the electrospinning solution has a great influence on the fiber diameter in electrospun gelatin fiber mats.

#### Effect of Solvent System on the Fiber Diameter

**Fig. 2:**
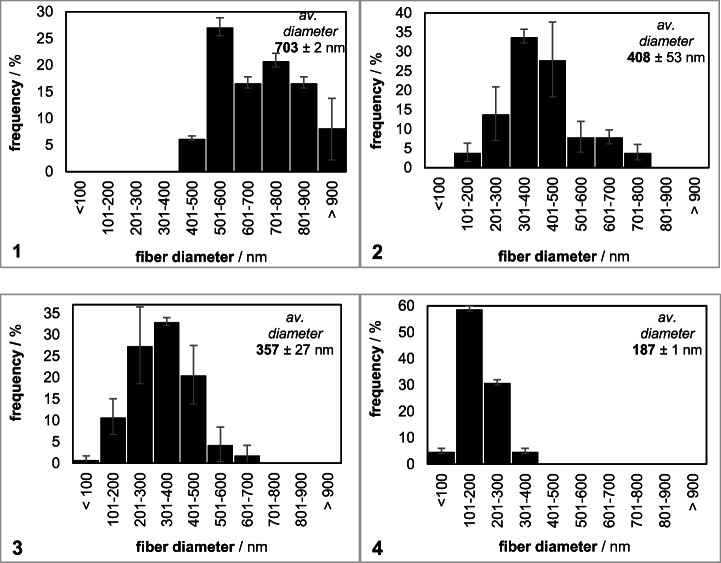
Diameter distribution and average diameters of gelatin fibers in electrospun nanofiber mats. The TFE/citrate-phosphate buffer ratio in the electrospinning solution was (1) 90:10, (2) 80:20, (3) 70:30, and (4) 60:40

To study the influence of water on the diameter distribution in GFM, the rate of citrate-phosphate buffer in the solvent system was increased gradually from 10 up to 40 wt.%. Figure 2 shows a distinct shift of the average fiber diameter from larger (> 400 nm) to smaller values (< 400 nm) with increasing water content in the TFE/citrate-phosphate buffer system. The advantage of bigger diameters is the higher stability of GFM during mechanical stress exposure, e.g., by using GFM in a stirred tank reactor. In contrast, the specific activity of immobilized β-gal on gelatin fiber mats electrospun from a 70:30 or a 60:40 TFE/citrate-phosphate buffer solvent system is higher because of a larger surface-to-mass ratio. To select the appropriate TFE/citrate-phosphate buffer ratio, the final enzymatic activity and the mechanical stability should be considered.

**Fig. 3:**
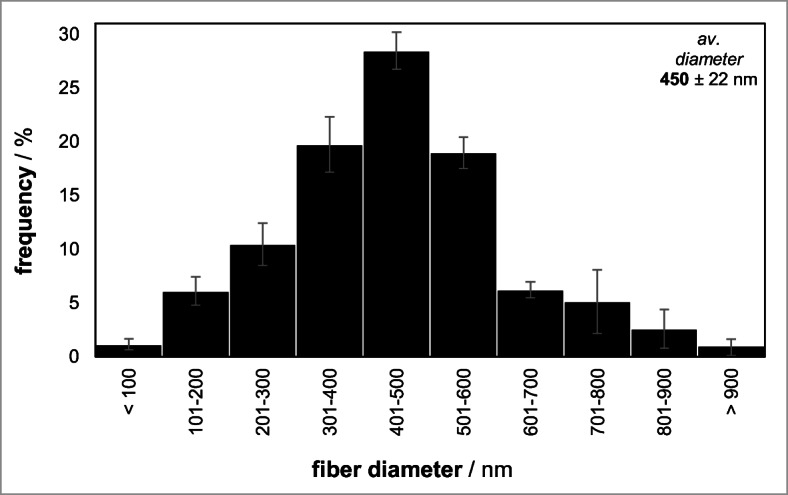
Diameter distribution in GFM produced via suspension electrospinning. Suspension electrospinning solution was gelatin [(10 wt.%)//TFE/0.02 M citrate-phosphate buffer (pH 4.5) (70:30)], and β-gal concentration in electrospinning solution was 0.027 wt.%

For the immobilization of β-gal via suspension electrospinning, Fig. 3 presents a clear increase of the average fiber diameter in suspGFM most likely due to implemented enzymes in the fibers. Moreover, the diameter distribution of suspGFM broadens to the entire diameter bandwidth from 100 to 900 μm.

#### Morphology of Electrospun GFM

**Fig. 4:**
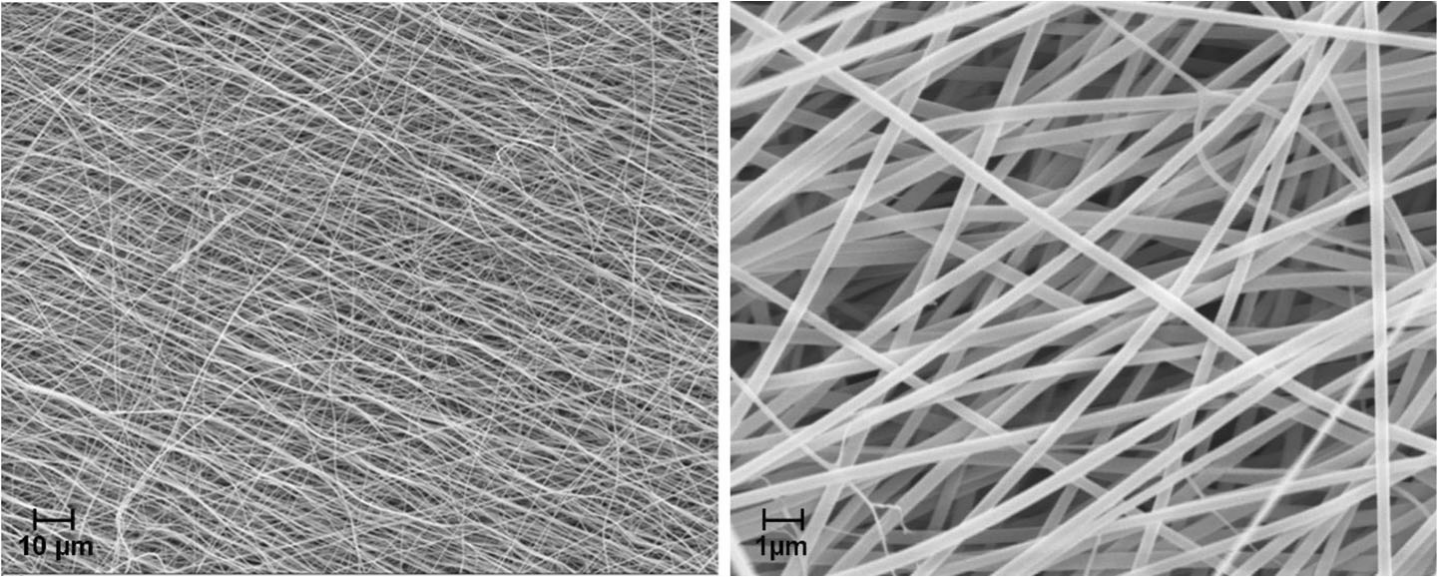
SEM images of electrospun gelatin fiber mats with a TFE/citrate-phosphate buffer solvent system [gelatin 10 wt.%//TFE/0.02 M citrate-phosphate buffer (pH 4.5) 70:30]. Magnification on the left is 500× and on the right is 5000×

The inspection of GFM under a scanning electron microscope shows that the gelatin fibers have an even structure with a smooth and consistent fiber surface without any pores or strong buckling (Fig. 4). The nanofibers are mostly aligned parallel and only in the minority possess a random orientation in a three-dimensional, entangled structure which overlays the parallel fibers. The nanofibers do not interact (e.g., bonding or conglutination) between each of the single filaments. Gelatin fiber mats are stable to touching and light pulling by hand.

### Influence of GFM Cross-Linking Time on the Activity of Immobilized β-Galactosidase

**Fig. 5:**
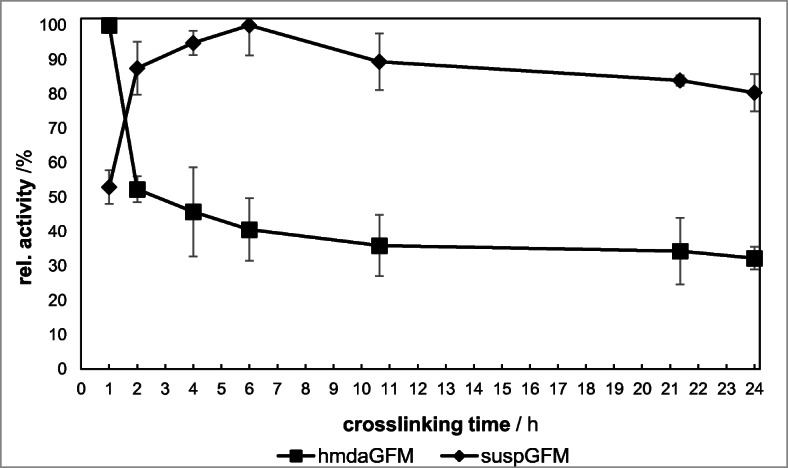
Dependence of enzymatic activity of immobilized β-gal on GDA exposure time

GFM are naturally not water-stable and already dissipate on contact with small amounts of water or single water droplets. In order to enhance their stability in water, GFM were stored under a glutaraldehyde atmosphere in a sealed glass container stimulating the linkage formation between surface amino groups of adjacent gelatin fibers. The activity of immobilized β-gal is dependent not only on the fiber diameter but also on the cross-linking time of the gelatin fiber mats under glutaraldehyde atmosphere (Fig. 5).

An increase of cross-linking time leads to a strong decrease of β-gal activity on hmdaGFM by 50% after 2 h and up to 70% after 24 h. It can be assumed that surface amino groups at long GDA reaction times are in large part no longer available for chemical bonding of β-gal to hmdaGFM due to the linkage of terminal amino groups with GDA. An advantage of longer GDA exposure times is the enhanced mechanical stability under stress conditions, which may compensate the β-gal activity reduction.

The activity of suspGFM initially rises continuously until reaching an activity maximum after 6 h. Entrapped enzymes are probably also covalently bound to the fiber via GDA, so that there is less loss of β-gal due to washing out. A further increase of cross-linking time (> 6 h) in turn probably causes an inactivation of β-gal.

### Immobilization Parameters

For the immobilization of β-gal onto GFM, it was most important, on one hand, to maximize the specific activity of β-gal on the fiber mats and, on the other hand, to design an immobilization procedure which maintains a constant enzyme activity over a long period.

#### Immobilization Parameters of β-gal on hmdaGFM

During evaluation of the hmdaGFM immobilization process, four parameters (temperature, pH, immobilization time, and initial β-gal concentration) were optimized. The activity of immobilized β-gal was determined via an oNPG assay.

**Fig. 6:**
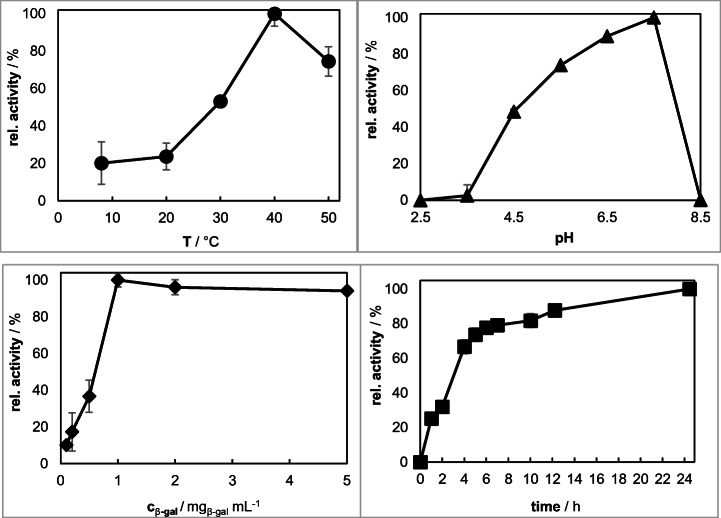
Progression of immobilization parameters of β-galactosidase onto hmdaGFM

Figure 6 illustrates that the specific activity of β-gal on hmdaGFM is strongly dependent on temperature, pH, immobilization time, and initial β-gal-concentration in the immobilization solution. An overview of the optimum immobilization parameters is shown in Table 1.

**Table 1:** Overview of the optimum immobilization parameters for hmdaGFM

Reaction time (h)	Temperature (°C)	pH	β-Gal concentration (mg/mL)
24.5	40	7.5	1

First, the effect of the temperature on the specific activity was studied by varying the immobilization temperature between 8 and 50 °C while other parameters were held at pH 4.5, 1 mg/mL β-gal concentration, and 24 h. A steep increase in activity was found when raising the temperature stepwise from 20 °C to 40 °C. The specific activity of β-gal rises continuously until reaching its maximum at 40 °C. At 50 °C, the activity falls back to 75% probably due to inactivation of free enzyme in the reaction medium at higher temperatures.

The pH also holds a major influence on the specific activity of immobilized β-gal. To investigate the impact of the pH on the immobilization, it was varied between 2.5 and 8.5 while other parameters were fixed at 40 °C, 1 mg/mL β-gal concentration and 24 h. There is a steady increase in specific activity until pH 7.5 with a subsequent strong drop to 0.15% at pH 8.5. This profile is contrary to the pH-profile of free β-gal (see Fig. 3) where 50% of the enzyme is already inactivated at pH 7.5. It is assumed that amino groups on the β-gal surface either better connect to GDA at higher pH values or that the mobile HMDA-GDA spacer on the hmdaGFM surface has an elevated stability at pH 7.5 in comparison to pH 4.5.

The initial β-gal concentration was varied from 0.1 to 5 mg/mL in order to study the influence of enzyme concentration on the specific activity. Other parameters were kept at 40 °C, pH 7.5, and 24 h. There is a steady increase in activity up to the maximum at 1 mg/mL, and the activity slightly decreases in the following development to around 95%. Higher amounts of β-gal probably lead to steric hindrances in substrate approach toward the enzyme.

As the final step in the parameter optimization, the immobilization time was varied between 0 and 24.5 h at fix parameter of 40 °C, 7.5, and 1 mg/mL β-gal concentration. An increase of the immobilization time up to 24.5 h leads to a higher specific activity on GFM.

#### Immobilization Parameters of β-gal on suspGFM

**Fig. 7:**
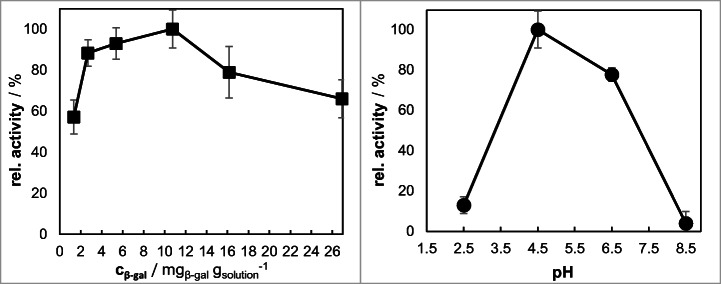
Overview of the immobilization parameters of β-galactosidase onto suspGFM. suspGFM was electrospun at 25 °C and 30% humidity from a 10 wt.% gelatin solution. suspGFM was cross-linked for 6 h with GDA

During the suspension electrospinning process, two main parameters were varied as follows: the β-gal concentration in the electrospinning solution was investigated between 1.3 and 26.9 mg_β-Gal_/g_solution_ and the pH of the citrate-phosphate buffer as part of the binary solvent system was varied from pH 2.5 to pH 8.5. The optima for the production of suspGFM are listed in Table 2. Figure 7 shows the course of the immobilization parameters.

**Table 2:** Overview of the optimum immobilization parameters for suspGFM

pH	β-Gal concentration (mg_β-gal_/g_solution_)
4.5	10.8

An increase of the β-gal concentration in the electrospinning solution correlates with the residual activity on suspGFM until reaching a maximum at 10.8 mg_β-gal_/g_solution_. The accumulation of β-gal on the fiber surface due to high initial concentration levels (> 10.8 mg_β-gal_/g_solution_) has an unfavorable effect of the overall activity of the enzyme. There is a reduction in specific activity to 79% at 16.2 mg_β-gal_/g_solution_ and finally to only 66% at 26.9 mg_β-gal_/g_solution_. The optimal β-gal concentration was found at 10.8 mg_β-gal_/g_solution_.

### pH and Temperature Effect on the Activity of Free and Immobilized β-Galactosidase

The dependence of the catalytic activity of free and immobilized β-galactosidase from *Aspergillus oryzae* on pH and temperature was studied. The enzyme activity was determined by an oNPG activity assay with pH ranging from 2.5 to 7.5 at a fixed temperature of 30 °C.

**Fig. 8:**
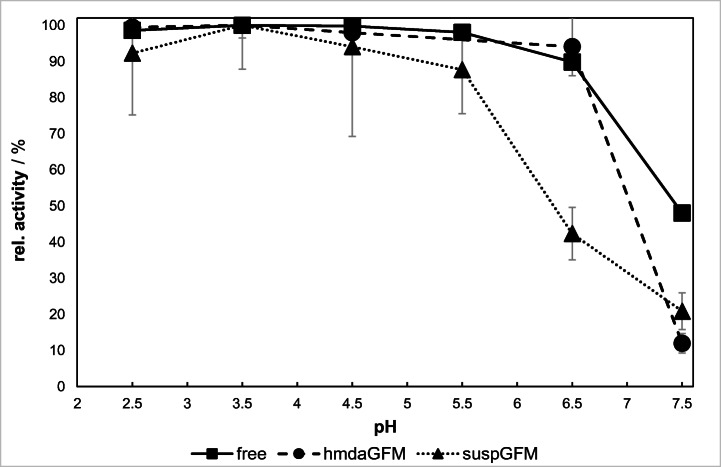
Relative activity of free and immobilized β-galactosidase at varied pH values (T = 30 °C)

There is a broad range of pH (2.5–5.5) where no significant changes in relative activity of β-gal can be observed (Fig. 8). At pH 6.5, the activity starts to decline to 90% and further drops quickly to only 48% at pH 7.5.

The activity of immobilized β-galactosidase on hmdaGFM demonstrates a similar pH profile in comparison to the free enzyme. The activity stagnates on an even level within the range of pH 2.5–6.5. A sudden decrease in activity down to 12% occurs at pH 7.5. Both the free enzyme and immobilized β-gal on hmdaGFM do not show a distinct activity maximum.

The activity progression of β-gal on suspGFM looks slightly different compared with the free enzyme and hmdaGFM. There is an apparent activity maximum at pH 3.5. With increasing pH, the activity decreases moderately to 21% at pH 7.5.

Slight deviations in pH characteristics of hmdaGFM compared with the free enzyme may be due to changes in the enzyme microenvironment. Changes are on the one hand induced by cross-linking of gelatin fibers with GDA whereby amino groups are transformed into imines. On the other hand, they are initiated by activating gelatin fibers with HMDA. Both processes lead to a less basic and less polar GFM surface. Furthermore, covalent bonding of β-gal to HMDA spacer molecules may cause modifications in the intrinsic activity of the enzyme or in the three-dimensional conformation. In addition, the active site, which consists of Glu200 as an acid/base catalyst and Glu298 as the nucleophile may be affected during immobilization [20]. As a result, the interaction between enzyme and substrate may be different from the bulk solution. β-Gal immobilized on suspGFM does not only underlie changes in the microenvironment because of gelatin fiber cross-linking but beyond that the enzymes are interconnected among each other which results in more visible changes in pH profile with a more narrow activity distribution in comparison to free or HMDA-immobilized β-gal.

**Fig. 9:**
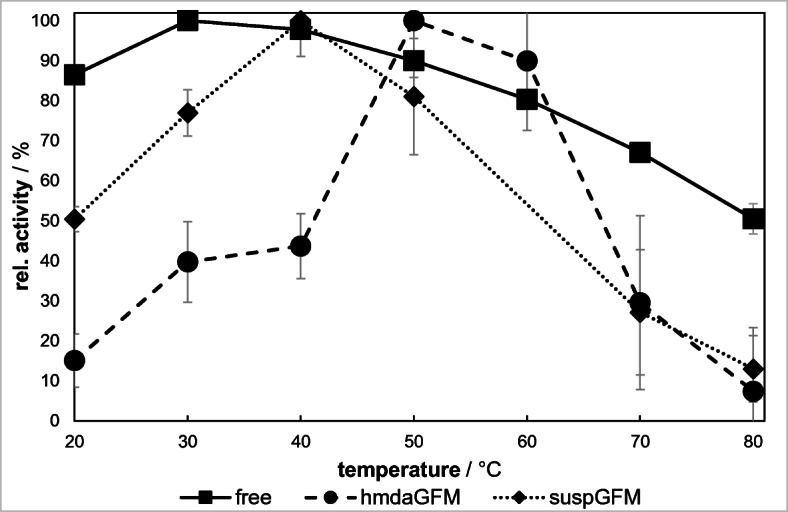
Dependence of the catalytic activity of β-galactosidase on the temperature (pH 4.5)

The impact of temperature on the activity of free and immobilized β-gal was analyzed by adjusting the temperature from 20 to 80 °C and keeping the pH fixed at 4.5 (free β-gal) and 3.5 (immobilized β-gal), respectively. The activity of β-gal was determined via oNPG assay. Figure 9 illustrates the course of the specific activity of β-gal at different temperatures in the reaction medium.

The activity of free β-gal reaches its maximum at 30 °C. On both sides of the maximum, the activity decreases steadily until reaching 50% of the maximum activity at 80 °C. The temperature maxima of immobilized β-gal are shifted to 40 °C (suspGFM) and 50 °C (hmdaGFM). On the left and right sides of the maxima, the loss of activity is more rapid compared with the free enzyme. At 70 °C, there is only 30%, and at 80 °C, there is only 7% of the original activity left.

### Operational Stability of Immobilized β-Galactosidase

The operational stability of immobilized β-gal on hmdaGFM and suspGFM was studied over a period of 28 days at 584 mmol/L lactose concentration, pH 3.5, and a temperature of 30 °C. Immobilized β-gal was placed in lactose solution, and the samples were withdrawn regularly at defined time intervals. Remaining activity was determined via a lactose activity assay. The first-order operational inactivation rate (k) was calculated from the following equation:

1 $$ \ln \mathrm{A}=\ln {\mathrm{A}}_0-\mathrm{k}\bullet \mathrm{t} $$

where lnA is the remaining activity after time *t* and *A*_0_ is the initial activity at *t* = 0.

The operational inactivation rate at 30 °C, pH 3.5, and 584 mmol/L lactose concentration for hmdaGFM was 9.36•10^−4^/h, and for suspGFM, it was calculated to be 7.44•10^−4^/h.

Both hmdaGFM and suspGFM preserve the enzymatic activity fairly well with 65% (hmdaGFM) and 81% (suspGFM) residual activity after 670 h of storage at process conditions. The half-life of immobilized β-gal is 740 h for hmdaGFM and 931 h for suspGFM, which verifies GFM as an excellent carrier material for β-gal in continuous GOS production processes.

### Kinetic Studies on Immobilized β-Galactosidase

The kinetic parameters were determined via oNPG activity assay by varying the oNPG concentration from 1 to 50 mmol/L at pH 4.5 and 30 °C (free β-gal) and pH 3.5, 40 °C for suspGFM and hmdaGFM. Both free and immobilized β-gal fitted the Michaelis–Menten equation with coefficients of determination (*R*^2^) of at least 90%.

The Michaelis–Menten parameters for free and immobilized β-gal are summed up in Table 3.

**Table 3:** Michaelis–Menten parameters of free β-gal, hmdaGFM, and suspGFM

	K_M_ (mmol/L)	v_max_	*R*^2^ (%)
Free β-gal	16.7 ± 0.63	16.0 ± 0.19 mmol/min/mg_β-gal_	90
hmdaGFM	7.8 ± 0.58	30.3 ± 0.09 mmol/min/g_hmdaGFM_	95
suspGFM	10.0 ± 0.60	568 ± 32.5 mmol/min/g_suspGFM_	94

Both K_M_ and v_max_ differ extensively from the literature values but conform to the manufacturers’ specifications [21, 22]. K_M_ of immobilized β-gal declines by one-third for suspGFM and more than half for hmdaGFM. suspGFM shows a much higher maximum velocity per gram than hmdaGFM, which makes them more attractive for fast processes.

### Product Inhibition by Galactose

Galactose is one of the products of lactose hydrolysis catalyzed by β-gal. It was reported previously that galactose has an inhibitory effect on all enzymatic reactions of β-gal [23–25]. To investigate the extent of the inhibition, galactose was varied between 28 and 555 mmol/L at a fixed lactose concentration of 140 mmol/L. Other parameters were adjusted to be pH 4.5, 30 °C (free β-gal) and pH 3.5, 40 °C (immobilized β-gal).

**Fig. 10:**
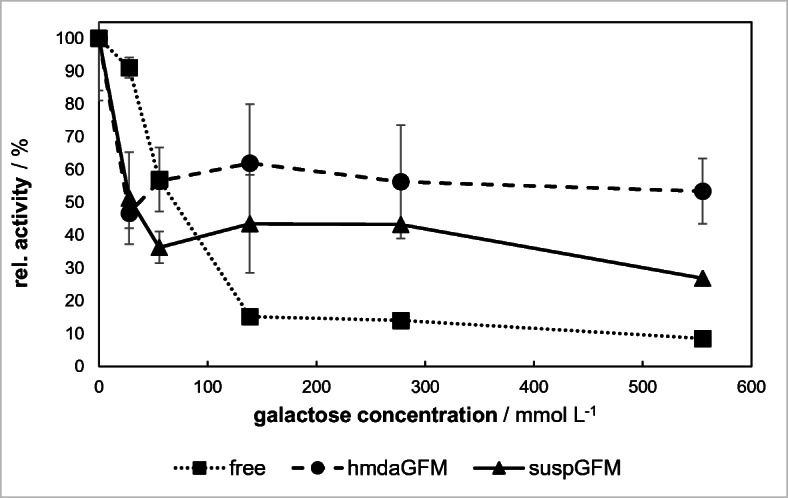
Influence of the galactose concentration on the activity of free and immobilized β-gal

The activity of free β-gal quickly decreases to only 15% at a galactose concentration of 140 mmol/L (Fig. 10). An ensuing quadruplication of the galactose concentration halves the β-gal activity to 8.5%. A separation of galactose from the reaction medium should be considered in the application of free β-gal.

The immobilization of β-gal significantly improves the activity with three- (suspGFM) and four-fold residual activity (hmdaGFM) at 140 mmol/L galactose concentration. A special course of the activity decrease was detected at lower (< 140 mmol/L) galactose concentrations, where an activity minimum is reached at 28 mmol/L (suspGFM) and 55 mmol/L (hmdaGFM).

### GOS Production

It is well-known that the initial lactose concentration has a major influence on the maximum GOS concentration. High initial lactose concentrations lead to higher GOS yields due to decreased water activity and an increased availability of enzymatic acceptors. Typical optimized yields range from 30% to 40% [26]*.*

hmdaGFM and suspGFM show a reduced activity in lactose solutions with relatively high viscosities (≥ 1.75 mol/L lactose). Therefore, the amount of GOS was quantified in a 1.17 mol/L lactose solution at pH 4.5 (free β-gal) and pH 3.5 (hmdaGFM, suspGFM) over a period of 7 days. Temperature for all tests was 30 °C. The total GOS amount and the degree of polymerization (DP) were analyzed by HPLC.

**Fig. 11:**
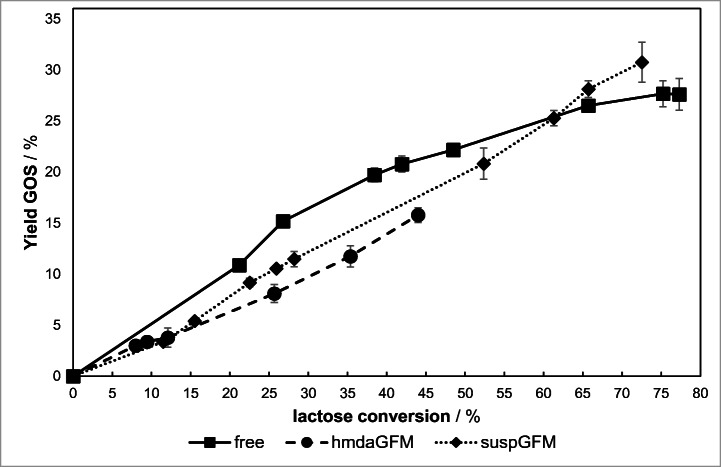
Total GOS yield as a function of the lactose conversion. Reaction was carried out at pH 4.5 (free β-gal) and pH 3.5 (immobilized β-gal), in 30 °C and 1.17 mol/L lactose concentration over a period of 7 days

The GOS course of free β-gal passes a maximum at 75% lactose conversion with a GOS yield of 27.7% in the reaction mixture (Fig. 11). The velocity of hmdaGFM is notably lower than the velocity of free β-gal, so only 44% of lactose is converted after 7 days. The structure of free β-gal and β-gal in suspGFM is most likely approximately identical due to the inclusion of the whole enzyme into the gelatin nanofibers. Because of covalent bonding of β-gal to gelatin nanofibers in hmdaGFM, the active site of the enzyme may be affected resulting in different GOS yields over time.

The suspGFM GOS yield reaches its peak at 72.5% lactose conversion with a yield of 31%. Thus, the GOS yield of suspGFM is 10% higher than the yield of the free enzyme. It could be assumed that the increased galactose concentration over time inhibits the free enzyme to a greater extend (see Fig. 10), so that the hydrolytic cleavage of lactose and GOS becomes the favored reaction.

**Fig. 12:**
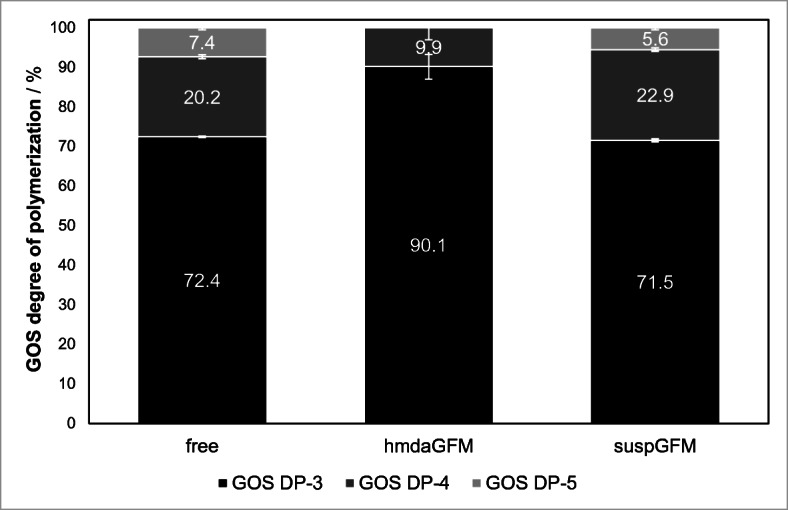
Composition of the GOS fraction after 7 days of reaction time (pH 4.5, 30 °C, 1.17 mol/L initial lactose concentration)

The GOS fraction of suspGFM and free β-gal almost has an identical composition with around 72% of DP-3, 21% of DP-4, and 5–7% of DP-5 (Fig. 12). The GOS fraction of hmdaGFM does not include DP-5, since the reaction did not make as much progress as the reaction of free β-gal and suspGFM. The ratio of DP-3 and DP-4 in the hmdaGFM reaction is 90% to 10%.

## Conclusion

β-Galactosidase from *Aspergillus oryzae* was successfully immobilized on electrospun gelatin fiber mats via covalent bonding to HMDA-activated mats and direct immobilization during the electrospinning process. Both hmdaGFM and suspGFM showed an excellent long-term operational stability. Therefore, they are suitable for continuous GOS production processes. Due to the simplicity of the method and a prompt usability of the fiber mats after their production, immobilization via suspension electrospinning is suggested for all rapid standard STR applications, which do not require very high stirring rates. The transgalactosylation activity of suspGFM was very well preserved, so a higher GOS yield could be achieved by suspGFM in comparison to the free enzyme.

Further studies are required to optimize the production of GOS in a suspGFM batch reactor and to understand the mechanism of GOS production completely.
